# Quantification of circulating cell-free DNA to predict patient survival in non-small-cell lung cancer

**DOI:** 10.18632/oncotarget.21769

**Published:** 2017-10-10

**Authors:** Myung Han Hyun, Jae Sook Sung, Eun Joo Kang, Yoon Ji Choi, Kyong Hwa Park, Sang Won Shin, Sung Yong Lee, Yeul Hong Kim

**Affiliations:** ^1^ Division of Medical Oncology, Department of Internal Medicine, Korea University Medical Center, Korea University College of Medicine, Seoul, Republic of Korea; ^2^ Cancer Research Institute, Korea University, Seoul, Republic of Korea; ^3^ Division of Pulmonary, Allergy and Critical Care Medicine, Department of Internal Medicine, Korea University Medical Center, Korea University College of Medicine, Seoul, Republic of Korea

**Keywords:** cell-free DNA concentration, prognostic value, non-small-cell lung cancer

## Abstract

We used computed tomography (CT) to explore the prognostic value of cell-free (cf) DNA quantification and its predictive efficacy over time after chemotherapy in non-small-cell lung cancer (NSCLC) patients. In total, 177 NSCLC patients were enrolled in a prospective biomarker trial. Consecutive paired blood collection was performed to determine cfDNA concentrations at baseline CT and throughout serial follow-ups. The best cfDNA cut-off value to predict progression-free and overall survival was determined using X-tile analysis. Among 112 chemo-naive patients with stage IV adenocarcinoma, 43 were available for follow-up analysis. Cox regression multivariate analysis indicated that a high cfDNA concentration was an independent negative prognostic factor for progression-free survival (hazard ratio: 2.60; 95% confidence interval: 1.65-4.10; p = 0.008) and overall survival (hazard ratio: 2.63; 95% confidence interval: 1.66-4.17; p < 0.001). However, cfDNA concentration changes during treatment did not correlate with radiological CT responses at first follow-up or best response. No pattern was noted in the percent change in the cfDNA concentration from baseline or subsequently measured level to progression. The serum cfDNA concentration is thus associated with NSCLC patient prognosis, but does not appear to be a clinically valid marker for tumor responses.

## INTRODUCTION

Traditional biopsy from the primary tumor site has limitations due to its invasive nature [1]. In particular, in lung cancer, which is among the leading causes of cancer-related death worldwide, obtaining a tumor tissue sample in the thoracic cavity to guide treatment decisions is challenging [2, 3]. Therefore, there is a high demand for continuous monitoring through detectable tumor surrogate specimens in blood with sensitive molecular biology techniques [4, 5]. Presently, there is a renewed interest in liquid biopsy using circulating cell-free deoxyribonucleic acid (cfDNA) because of its potential to screen for tumors and disease progression in lung cancer [6].

Tumor cells can release genomic DNA into the bloodstream through numerous mechanisms, such as leakage after tumor necrosis or apoptosis, lysis of circulating tumor cells during micrometastasis, and macrophage digestion of tumor DNA fragments [7, 8]. Theoretically, the amount of tumor-derived cfDNA should correlate with the tumor burden or biological aggressiveness in malignant conditions, due to both passive and active release mechanisms [9, 10]. Previous clinical trials have demonstrated that the amount of cfDNA is higher in patients with lung cancer than in healthy individuals [11, 12]. In addition, high baseline cfDNA concentrations have been found to worsen the survival outcomes of patients with lung cancer [13, 14]. However, relatively few studies have investigated changes in cfDNA levels at specific periods during treatment [15-21]. To date, detailed analyses of dynamic changes in the cfDNA concentration to predict the radiological tumor response have not been performed. Despite the growing interest in cfDNA as a potential biomarker for lung cancer, the clinical validity of cfDNA quantification remains inconclusive.

In mid-2016, the first liquid biopsy test was approved by the U.S. Food and Drug Administration [22, 23]. The concentration of tumor cfDNA, which is known to have a remarkably short half-life (∼16 min), can be determined simply by liquid biopsy to predict clinical disease progression and patient survival [24]. Therefore, in this study, we explored the prospective predictive and prognostic value of cfDNA quantification over time with computed tomography (CT) imaging after chemotherapy in patients with non-small-cell lung cancer (NSCLC).

## RESULTS

### cfDNA concentration and clinical characteristics

The correlations of patients’ clinicopathologic characteristics with their baseline cfDNA concentrations are shown in Table 1. In total, 177 patients (115 [65%] men and 62 [35%] women) with baseline cfDNA data were included in the study, with a median age of 65 years. Among these patients, 112 (67 [60%] men and 45 [40%] women) chemo-naive patients with stage IV adenocarcinoma were analyzed in the follow-up cohort, with the same median age. In both data sets, most of the patients had a good performance status (Eastern Cooperative Oncology Group performance status [ECOG PS] ≤ 1), but also had high comorbidity (Charlson Comorbidity Index > 7). Most patients in the total population had advanced-stage (stage IV, 77%) adenocarcinoma (79%) without previous chemotherapy before sampling (95%). A greater number of patients had bone metastasis than liver metastasis in both the total and chemo-naive stage-IV adenocarcinoma-only patient populations (45% vs. 15% in total patients; 63% vs. 20% in chemo-naive stage-IV adenocarcinoma-only patients).

**Table 1:** Correlation of baseline circulating cfDNA amounts with clinicopathologic characteristics in patients with NSCLC

Variables	All patients with NSCLC (n = 177)			Chemo-naive patients with stage IV ADC (n = 112)		
	Patients, n (%)	cfDNA amount (ng/mL)^*^	p value	Patients, n (%)	cfDNA amount (ng/mL)^*^	p value
Age						
< Median	82 (46)	64 (33–120)		55 (49)	72 (38–135)	
> Median	95 (54)	57 (39–139)	0.938	57 (51)	56 (39–157)	0.354
Sex						
Male	115 (65)	57 (37–139)		67 (60)	66 (38–145)	
Female	62 (35)	59 (40–115)	0.817	45 (40)	60 (40–133)	0.973
ECOG status						
≤ 1	149 (84)	56 (37–133)		90 (80)	61 (38–147)	
> 1	28 (16)	73 (41–129)	0.192	22 (20)	73 (40–120)	0.299
Charlson Comorbidity Index						
≤ 7	50 (28)	50 (30–110)		14 (12)	110 (48–276)	
> 7	127 (72)	64 (39–139)	0.283	98 (88)	59 (38–132)	0.116
Histology type^**^						
ADC	140 (79)	56 (35–130)		112 (100)	63 (39–141)	
SQCC	26 (15)	67 (44–119)		0 (0)	N/A	
Others	11 (6)	63 (40–286)	0.538	0 (0)	N/A	N/A
EGFR mutation						
Yes	77 (49)	56 (40–135)		61 (57)	57 (38–135)	
No	79 (51)	63 (39–133)	0.268	46 (43)	75 (39–142)	0.467
Previous chemotherapy before sampling						
Yes	9 (5)	58 (31–147)		0 (0)	N/A	
No	168 (95)	59 (31–133)	0.574	112 (100)	63 (39–141)	N/A
Clinical stage^***^						
Stage I/II	18 (10)	41 (25–46)		0 (0)	N/A	
Stage III	22 (13)	67 (41–87)		0 (0)	N/A	
Stage IV	137 (77)	72 (41–159)	0.003	112 (100)	63 (39–141)	N/A
Liver metastasis						
Yes	27 (15)	66 (34–115)		22 (20)	69 (29–112)	
No	150 (85)	57 (38–134)	0.511	90 (80)	61 (41–149)	0.575
Bone metastasis						
Yes	81 (45)	71 (39–157)		71 (63)	69 (39–141)	
No	96 (55)	51 (32–113)	0.084	41 (37)	56 (39–140)	0.366

cfDNA, cell-free DNA; NSCLC, non-small-cell lung cancer; ADC, adenocarcinoma; ECOG, Eastern Cooperative Oncology Group; SQCC, squamous cell carcinoma; EGFR, epidermal growth factor receptor

^*^Data are expressed as the median, followed by the interquartile range in parentheses.

^**^According to the World Health Organization (WHO) classification [37]

^***^According to the American Joint Committee on Cancer (AJCC) 8th edition guidelines [38]

In brief, the cfDNA concentration did not correlate with baseline demographics, with the exception of clinical stage. In the total population, the cfDNA concentration was higher in patients with stage IV disease (median = 72 [interquartile range (IQR) = 41-159] ng/mL) than in those with stage III (median = 67 [IQR = 41-87] ng/mL) or stage I/II disease (median = 41 [IQR = 25-46] ng/mL) (p = 0.003). Receiver operating characteristic (ROC) curve analysis was performed to determine the power of cfDNA to discriminate patients with clinical stage IV disease from those with stage I-III disease. The results indicated that an area under the curve (AUC) of 0.658 (p < 0.002; 95% confidence interval [CI]: 0.568-0.748) had 62.0% sensitivity and 67.5% specificity to discriminate stage IV patients (Supplementary Figure 1).

### cfDNA concentration and patient survival

**Figure 1:**
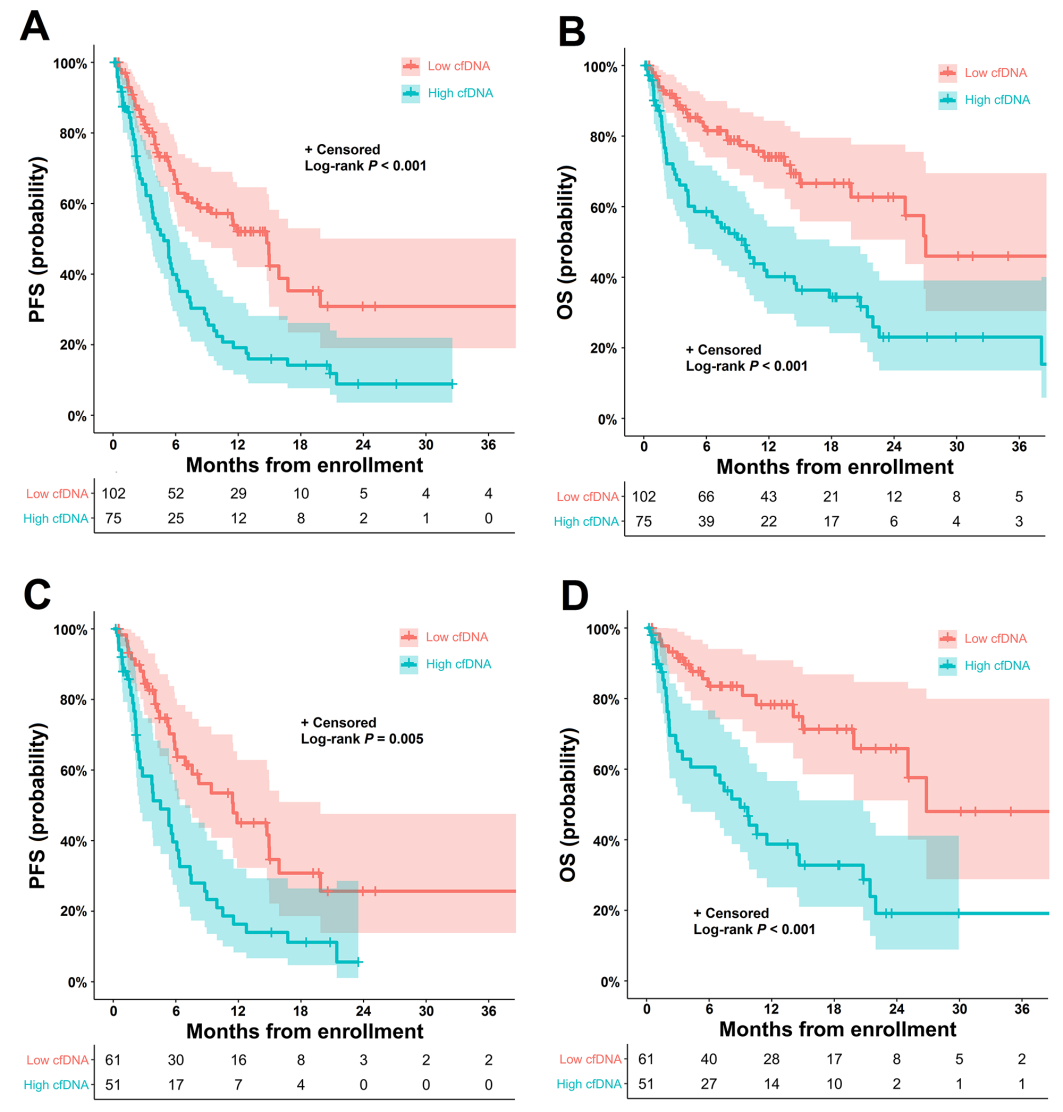
Kaplan-Meier estimates of PFS and OS according to the cfDNA concentration in patients with NSCLC **(A)** PFS and **(B)** OS according to the baseline cfDNA concentration (≤ 70 ng/mL vs. > 70 ng/mL) in all patients with NSCLC. **(C)** PFS and **(D)** OS according to the baseline cfDNA concentration (≤ 70 ng/mL vs. > 70 ng/mL) in chemo-naive patients with stage IV adenocarcinoma. cfDNA, cell-free DNA; NSCLC, non-small-cell lung cancer; PFS, progression-free survival; OS, overall survival.

The median progression-free survival (PFS) was 7.6 months (95% CI: 5.1-10.1 months), and overall survival (OS) was 22.2 months (95% CI: 15.2-29.2 months) in the total population. In chemo-naive patients with stage IV adenocarcinoma, the median PFS was 6.5 months (95% CI: 4.6-8.6 months) and OS was 22.2 months (95% CI: 14.1-30.3 months). Figure 1 displays the Kaplan-Meier plots of the PFS and OS probabilities in patients with high (> 70 ng/mL) and low (≤ 70 ng/mL) baseline cfDNA concentrations (70 ng/mL as the cut-off value). In the total population (Figure 1A and 1B), patients with high cfDNA concentrations had significantly shorter PFS (median PFS = 5.6 months; 95% CI: 4.0-7.3 months) and OS (median OS = 10.4 months; 95% CI: 6.5-14.2 months) than those with low cfDNA concentrations (median PFS = 15.8 months; 95% CI: 10.8-20.7 months; median OS = 28.9 months; 95% CI: 5.2-52.8 months) (all p value < 0.001). Similarly, in chemo-naive patients with stage IV adenocarcinoma (Figure 1C and 1D), high baseline cfDNA levels had a strong negative prognostic impact on PFS (low cfDNA vs. high cfDNA: median = 12.2 months [95% CI: 6.8-17.6 months] vs. median = 5.7 months [95% CI: 3.3-8.0 months], respectively; p = 0.005) and OS (low cfDNA vs. high cfDNA: median = 28.7 months [95% CI: 33.3-64.6 months] vs. median = 10.3 months [95% CI: 6.4-14.3 months], respectively; p < 0.001).

In univariate analysis, the cfDNA concentration, ECOG PS, Charlson comorbidity index and clinical stage were found to be significantly associated with PFS and OS in the total patient population. Multivariate Cox regression modeling identified a high cfDNA concentration as an independent negative prognostic factor for PFS (hazard ratio [HR]: 2.60; 95% CI: 1.65-4.10; p = 0.008) and OS (HR: 2.63; 95% CI: 1.66-4.17; p < 0.001). In chemo-naive patients with stage IV adenocarcinoma, a high cfDNA concentration was also an independent negative prognostic factor for PFS (HR: 2.21; 95% CI: 1.37-3.55; p = 0.001) and OS (HR: 3.50; 95% CI: 1.90-6.45; p < 0.001) (Table 2).

**Table 2:** Univariate and multivariate Cox regression model for PFS and OS based on the cfDNA concentration in patients with NSCLC

Variables		Univariate analysis				Multivariate analysis			
		PFS		OS		PFS		OS	
	n/N (%)	HR (95% CI)	p value	HR (95% CI)	p value	HR (95% CI)	p value	HR (95% CI)	p value
All patients with NSCLC									
cfDNA (>70 ng/mL)	75/177 (42)	2.17 (1.47–3.20)	< 0.001	2.60 (1.65–4.10)	< 0.001	2.60 (1.65–4.10)	< 0.001	2.63 (1.66–4.17)	< 0.001
Age (> median)	95/177 (54)	1.03 (0.70–1.52)	0.856	1.42 (0.90–2.23)	0.126	0.95 (0.62–1.44)	0.808	1.05 (0.64–1.71)	0.836
Sex (male)	115/177 (65)	1.00 (0.67–1.50)	0.979	1.47 (0.90–2.39)	0.117	1.07 (0.71–1.63)	0.727	1.48 (0.90–2.46)	0.120
ECOG status (>1)	28/177 (16)	2.10 (1.25–3.52)	0.005	2.71 (1.59–4.63)	< 0.001	1.89 (1.09–3.28)	0.023	2.64 (1.48–4.69)	0.001
Charlson Comorbidity Index (>7)	127/177 (72)	1.97 (1.22–3.19)	0.006	2.23 (1.19–3.70)	0.010	0.99 (0.51–1.91)	0.989	1.21 (0.58–2.52)	0.601
Stage IV	137/177 (77)	2.68 (1.54–4.66)	< 0.001	2.33 (1.25–4.35)	0.007	2.29 (1.09–4.77)	0.027	1.88 (0.86–4.14)	0.113
Stage IV chemo-naive ADC only									
cfDNA (>70 ng/mL)	50/112 (45)	2.11 (1.32–3.37)	0.008	3.08 (1.71–5.57)	< 0.001	2.21 (1.37–3.55)	0.001	3.50 (1.90–6.45)	< 0.001
Age (> median)	57/112 (51)	1.07 (0.67–1.70)	0.774	1.51 (0.85–2.68)	0.154	1.10 (0.64–1.89)	0.722	1.30 (0.68–2.48)	0.427
Sex (male)	67/112 (60)	0.96 (0.60–1.55)	0.888	1.49 (0.82–2.71)	0.187	1.12 (0.66–1.90)	0.653	1.76 (0.89–3.45)	0.101
ECOG status (>1)	22/112 (20)	1.69 (0.95–3.00)	0.073	2.53 (1.35–4.73)	0.004	1.92 (1.03–3.59)	0.040	3.02 (1.49–6.13)	0.002
Charlson Comorbidity Index (>7)	98/112 (88)	0.70 (0.37–1.35)	0.295	1.09 (0.49–2.46)	0.820	0.56 (0.26–1.23)	0.152	0.72 (0.27–1.96)	0.534

PFS, progression-free survival; OS, overall survival; NSCLC, non-small-cell lung cancer; HR, hazard ratio; CI, confidence interval; cfDNA, cell-free DNA; ECOG, Eastern Cooperative Oncology Group; ADC, adenocarcinoma

### cfDNA concentration kinetics and radiological tumor responses

**Figure 2:**
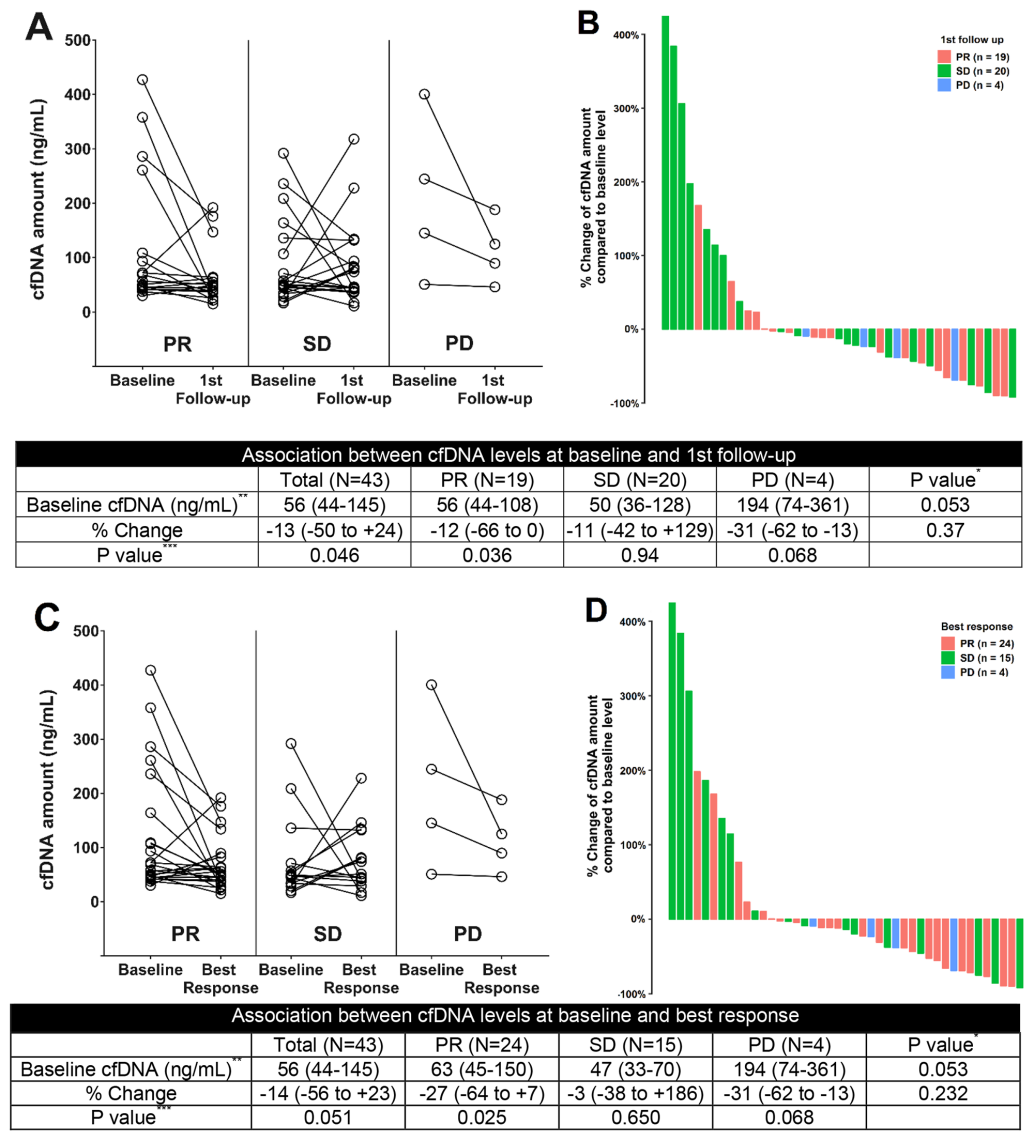
Changes in the cfDNA concentration during systemic therapy **(A)** Change in the cfDNA concentration from baseline to first response assessment, according to the radiological response category. **(B)** Waterfall plot for the percentage change in the cfDNA concentration at first response assessment. **(C)** Change in the cfDNA concentration from baseline to the radiological best response, according to the radiological best response category. **(D)** Waterfall plot for the percentage change in the cfDNA concentration at assessment of radiological best response. cfDNA, cell-free DNA; NSCLC, non-small-cell lung cancer; PR, partial response; SD, stable disease; PD, progression of disease ^*^Kruskal-Wallis test among PR, SD and PD groups ^**^Data are expressed as the median, followed by the interquartile range in parentheses ^***^Wilcoxon signed rank test between the cfDNA concentration at baseline and at first follow-up assessment or best response.

Measurable target lesions and adequate cfDNA for calibration were available for 43 patients who were enrolled for cfDNA kinetic analysis. The median follow-up period was 14.4 months. During first-line chemotherapy, the change in the cfDNA concentration is hypothesized to correlate with the radiological tumor response at different time points. In the partial response (PR) group only, the cfDNA concentration was lower at the first follow-up assessment than at baseline (median = −12% [IQR = −66% to +0%], p = 0.036) (Figure 2A). In the same group, the cfDNA concentration was significantly lower at the time point of the radiological best response than at baseline (median = −27% [IQR = −64% to +7%], p = 0.025) (Figure 2B). At both time points, however, no significant percentage changes in the cfDNA concentration were noted in the other response category groups. The waterfall plots also revealed no distinguishable pattern for the percentage change in the cfDNA concentration at assessments of first response and radiological best response (Figure 2C and 2D). In Spearman’s correlation analysis, the percentage change in the cfDNA level was not associated with the log-transformed percentage change in the sum of the longest diameter (SLD; r = 0.187, p = 0.223) (Supplementary Figure 2).

**Figure 3:**
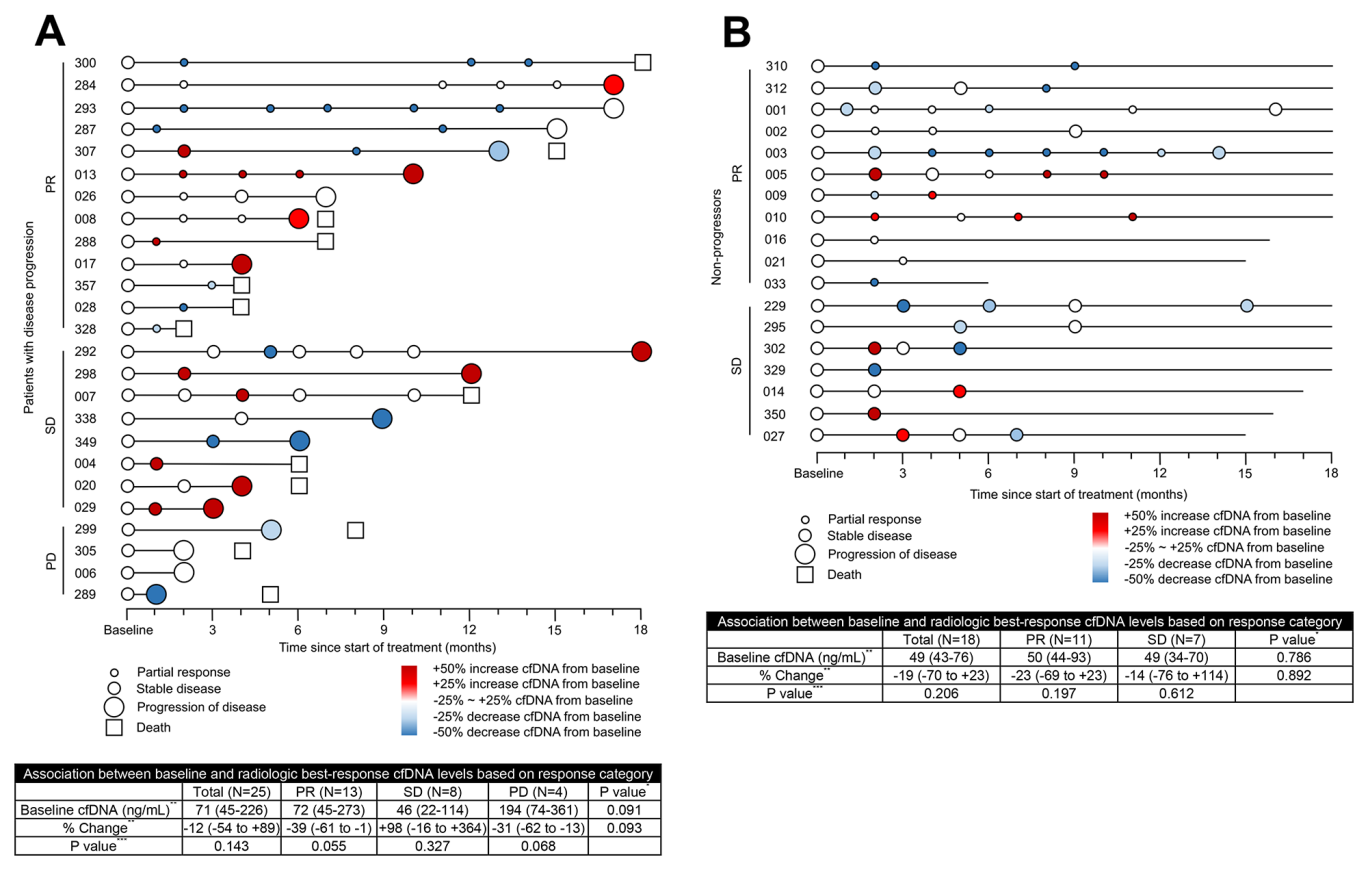
Circulating cfDNA time points coded by NSCLC patient identification number Graphical presentation of the association between the cfDNA level and the assessment of radiological response in patients with disease progression **(A)** and without progression **(B)** Change in the cfDNA concentration from baseline to best response, according to the radiological best response category; x-axis displays the time to clinical tumor progression. cfDNA, cell-free DNA; NSCLC, non-small-cell lung cancer; PR, partial response; SD, stable disease; PD, progression of disease ^*^Kruskal-Wallis test among PR, SD and PD groups ^**^Data are expressed as the median, followed by the interquartile range in parentheses ^***^Wilcoxon signed rank test between the cfDNA concentration at baseline and at best response.

Figure 3 is a graphical diagram of the association between the cfDNA concentration and the radiological response in patients with (n = 25 [58%]) and without disease progression (n = 18 [42%]). Of the 25 patients who experienced clinical progression after chemotherapy, 13 patients with PR, 8 patients with stable disease (SD) and 4 patients with progression of disease (PD) were identified based on their radiological best response during treatment (Figure 3A). Among the 13 patients in the PR group, 7 (54%) had a decrease in cfDNA concentration of more than 25% from baseline to best response. Of the 18 patients without disease progression, 11 patients belonged to the PR group and 7 belonged to the SD group (Figure 3B). Among the 11 patients in the PR group, 5 (45%) had a decrease in cfDNA concentration of more than 25% from baseline to best response. The percentage change in the cfDNA concentration at the time point of best response was hypothesized to differ according to the response category. However, no differences in the cfDNA concentration according to the response category were noted in patients either with disease progression (p = 0.093) or without progression (p = 0.892).

**Figure 4:**
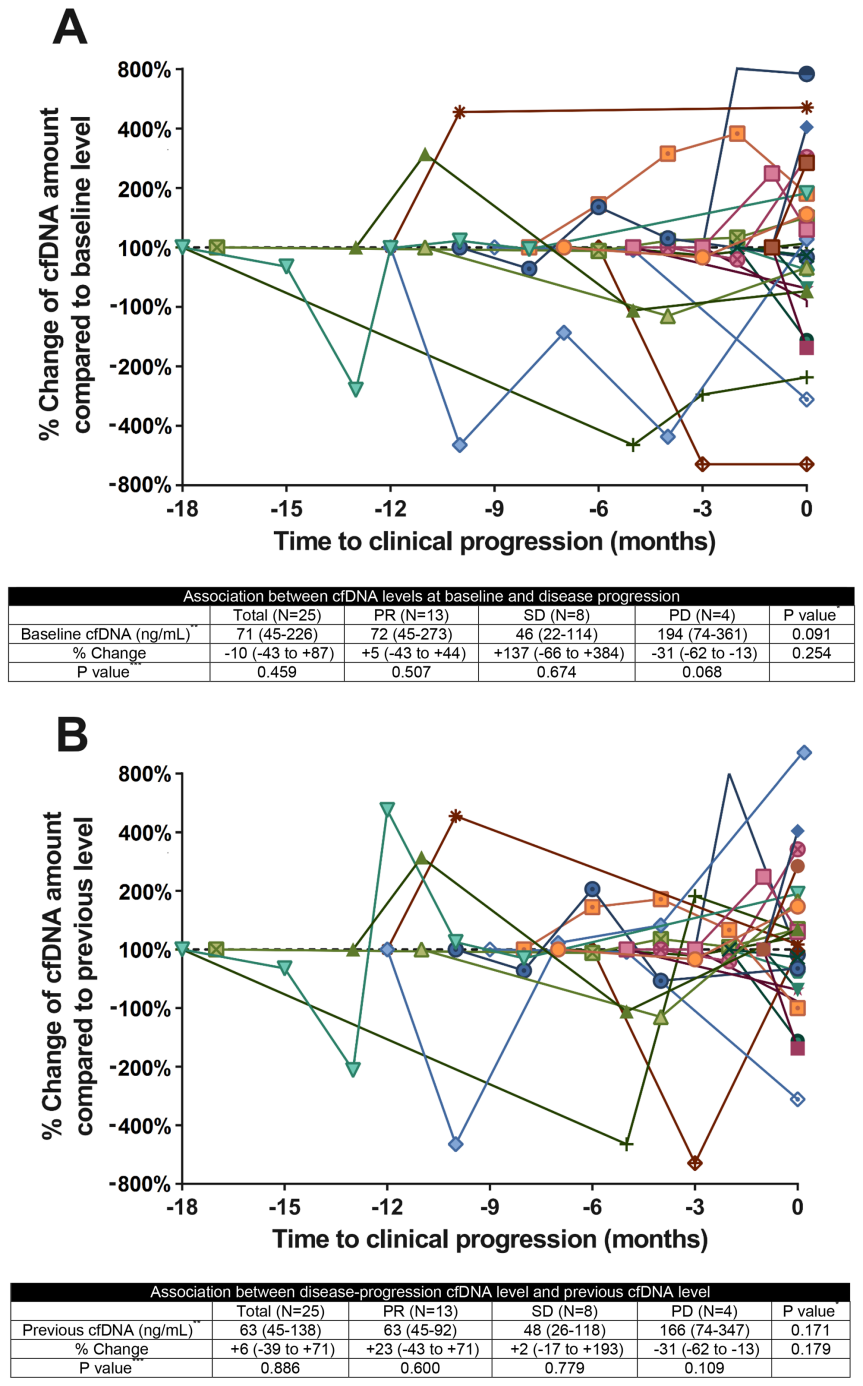
Circulating cfDNA kinetics in patients with NSCLCQuantitative cfDNA dynamics during treatment for NSCLC **(A)** Change in the cfDNA concentration from baseline to disease progression, according to the radiological best response category. **(B)** Change in the cfDNA concentration from the previous level to disease progression, according to the radiological best response category. Colors and symbols in the panel represent individual patient cfDNA kinetics; x-axis displays the time to clinical tumor progression. cfDNA, cell-free DNA; NSCLC, non-small-cell lung cancer; PR, partial response; SD, stable disease; PD, progression of disease ^*^Kruskal-Wallis test among PR, SD and PD groups^**^Data are expressed as the median, followed by the interquartile range in parentheses.^***^Wilcoxon signed rank test between the cfDNA level at disease progression and the baseline or previous cfDNA level.

Figure 4 depicts the kinetic pattern of the cfDNA concentration before disease progression. In the 25 patients with disease progression, no pattern was detected in the change in the cfDNA concentration from baseline and pre-progression measurements to progression. At the time point of disease progression, the percentage change in the cfDNA concentration from the baseline cfDNA concentration did not differ according to the radiological best response (p = 0.254) (Figure 4A). In addition, the percentage change in cfDNA between disease progression and the previous level did not differ according to the radiological best response (p = 0.179) (Figure 4B).

## DISCUSSION

With the recent advances in highly sensitive and specific quantitative analysis of blood cfDNA, measuring the cfDNA concentration is suggested to be a simple method to estimate the disease prognosis and tumor response [25, 26]. The first tumor-derived cfDNA in blood was identified in the 1970s, and the prognostic value of the cfDNA concentration was first reported in 1997 by Fournie et al., who analyzed 68 patients with NSCLC and small-cell lung cancer [27, 28]. In 2004, Gautschi et al. provoked greater interest in this field with their publication [15] describing 185 patients with NSCLC (stage I to IV). These authors reported that OS was significantly poorer for patients with high cfDNA concentrations than for those with low cfDNA concentrations (HR: 2.325) [15]. To date, numerous studies have demonstrated that the cfDNA concentration is related to survival in lung cancer [14, 19, 20, 29]. A recent meta-analysis of 17 studies with 1,723 patients indicated that OS was worse in patients with high cfDNA concentrations than in those with low cfDNA concentrations (HR: 1.76; 95% CI: 1.38-2.25; p < 0.001) [14]. Consistently, our multivariate Cox hazards model demonstrated that OS was worse (HR: 2.63; 95% CI: 1.66-4.17; p < 0.001) in patients with high cfDNA concentrations after adjustment for various clinical factors [15-18].

In contrast to the survival prognostic value, the predictive value of the cfDNA concentration for determining the radiological tumor response has been investigated in relatively few studies to date. The first study on this topic was published in 2004 by Gautschi et al. [15]. In that study, 91 patients with stage I-IV NSCLC underwent follow-up after the first to third cycles of chemotherapy, and the cfDNA concentrations at follow-up were compared to those at baseline. The cfDNA concentration was significantly elevated after chemotherapy in the PD group (p = 0.006), but not in the PR/SD group (p = 0.423). In addition, Pan et al. reported that the plasma DNA concentration was significantly reduced after the first cycle of chemotherapy in the PR group (p < 0.001), but not in the SD (p = 0.322) and PD groups (p = 0.528) among 82 patients with stage III/IV NSCLC [18]. Similarly, in an analysis of 40 patients with stage III/IV NSCLC, Vinayanuwattikun et al. reported an increasing trend in the cfDNA concentration 3 weeks after chemotherapy in the SD/PD group (p = 0.02) but not in the PR group [21]. However, these studies have had discordant results in different response groups, so it has not been possible to discriminate the response patterns among patients with PR, SD and PD. Similarly, Lee et al. and Tissot et al. could not use the change in the cfDNA concentration to differentiate patient response groups (p = 0.825, p = 0.473, respectively) [19, 20]. In an analysis of 42 patients with stage III-IV NSCLC, Kumar et al. reported that the cfDNA level after the third cycle of chemotherapy was significantly higher in the PD group than in the PR (p < 0.001) and SD (p = 0.001) groups [16]. However, the authors only compared cfDNA concentrations at the time of follow-up according to the response category, which cannot be generalized as the percentage change in cfDNA in individual patients.

Recently, Li et al. evaluated this issue in a large prospective study that included 103 patients with stage III/IV NSCLC [17]. Although the authors reported a weak monotonically increasing relationship between the percentage change in the cfDNA concentration and the SLD (Spearman’s correlation test, r = 0.21; p = 0.03), the percentage change in the cfDNA concentration from baseline to the first response evaluation period did not differ significantly among patients in different radiological response categories (Kruskal-Wallis test; p = 0.10) [17]. Nonetheless, the authors only investigated the first follow-up period and did not perform DNA kinetic analysis. Therefore, dynamic changes in the cfDNA concentration with the CT response require further clarification.

In this large prospective biomarker trial, we demonstrated that a high baseline cfDNA concentration was associated with worse PFS and OS in NSCLC patients. Automatic X-tile software was used to define the optimal cut-off value through training/validation methods, because result bias has occurred in previous studies employing median values, tertiles or ROC curves to define cut-off thresholds for the cfDNA concentration. In this study, we conducted numerous novel analyses that previous studies did not include. First, we used individual variation plots and waterfall plots to describe the change in the cfDNA concentration at different time points, including first response and best response, according to the radiological response category. Furthermore, we graphically presented the change in the cfDNA concentration corresponding to the radiological response for each patient at each assessment of radiological response. Lastly, we performed kinetic analysis during treatment to compare the cfDNA concentration at disease progression with baseline and previous measurements. In our analysis at various time points, however, the change in the cfDNA concentration during treatment did not correlate with the radiological CT response. The cfDNA kinetic analysis also revealed no distinguished pattern throughout the timeline.

Our findings can be explained through the following. First, high baseline levels of cfDNA may be associated with a high tumor burden and thus correlate with poor survival outcomes. The more the tumor cells enhance tumor apoptosis or other active releasing mechanisms, the higher the cfDNA levels will be in the blood. Additionally, aggressive tumors infiltrate normal tissue and increase necrosis, which is among the passive mechanisms known to release tumor-derived cfDNA into the blood [10]. Recently, Nygaard et al. investigated the correlation of the cfDNA concentration with the metabolic tumor volume and total lesion glycolysis using ^18^F-fluorodeoxyglucose positron emission tomography [30]. However, the authors concluded that the cfDNA concentration was not significantly associated with the metabolic tumor volume or total lesion glycolysis. Accordingly, the relationship between the cfDNA concentration and tumor burden requires further study. In addition, our study demonstrated that a high cfDNA concentration was associated with an advanced clinical stage (stage IV vs. stage I-III; AUC = 0.658, p < 0.002). Consistent with the results of previous research [31], an advanced clinical stage and a high tumor burden may contribute to the poor prognosis of patients with high concentrations of cfDNA.

Secondly, the cfDNA concentration cannot be used as a marker to predict the tumor response according to CT imaging. Though the exact mechanism is difficult to explain, we assume that beyond simple tumor lysis, more complex biological mechanisms underlie the variations in cfDNA levels. One explanation is that radiological assessment by CT may not reflect the true residual tumor burden, due to the difficulty of distinguishing cancer cells from surrounding tissue reactions. In addition, since cfDNA has a short half-life in blood (from approximately 15 min to a couple of hours), the cfDNA concentration may be altered prior to radiological changes during chemotherapy [32, 33]. Serial functional images coupled with timely analyses of patients’ tumor-drawn cfDNA concentrations are needed to elucidate this further.

Thirdly, the cfDNA concentration may be influenced by various factors, which must be considered in the interpretation of our results. Chemotherapy can induce apoptosis of both normal and malignant cells, thus aberrantly amplifying cfDNA levels in the circulation within 48 hours [8, 24]. Although the half-life of cfDNA in the circulation is short, transient variations in cfDNA levels may be a side effect of chemotherapy, and could confound the interpretation of the results [33]. In the present study, patients with severe side effects such as pulmonary embolism, trauma or other immunologically mediated diseases were excluded from the follow-up data set, and blood samples were collected more than 5 days after chemotherapy to reduce potential bias. Furthermore, the quality of the cfDNA purification process can impact the results. To optimize the preanalytical procedures, we limited the time gap (within 1 hour) to prevent the possible degradation of cfDNA [34]. Professional technicians gathered the blood samples immediately after these samples were obtained in the outpatient department or ward, then separated the supernatant from the cellular fraction using laboratory centrifuges located in the same center [35, 36]. In addition, to minimize DNA loss and improve the cfDNA yield, cfDNA was purified repeatedly to minimize contamination of the DNA. Accordingly, DNA extraction and PCR amplification were performed to obtain a more reliable quantitative measurement of cfDNA.

In conclusion, our findings suggest that the serum cfDNA concentration is associated with the prognosis of patients with NSCLC. However, our results do not support the clinical validity of cfDNA as a predictive biomarker for tumor responses. Further large routine-based clinical trials for cfDNA kinetics are required to confirm our results.

## MATERIALS AND METHODS

### Ethical statement

This study was conducted in accordance with the declaration of Helsinki, following approval from the Institutional Review Board of Korea University Medical Center (ED14110). Patients provided written consent for their information to be stored in the hospital database and used for research.

### Study population and design

**Figure 5:**
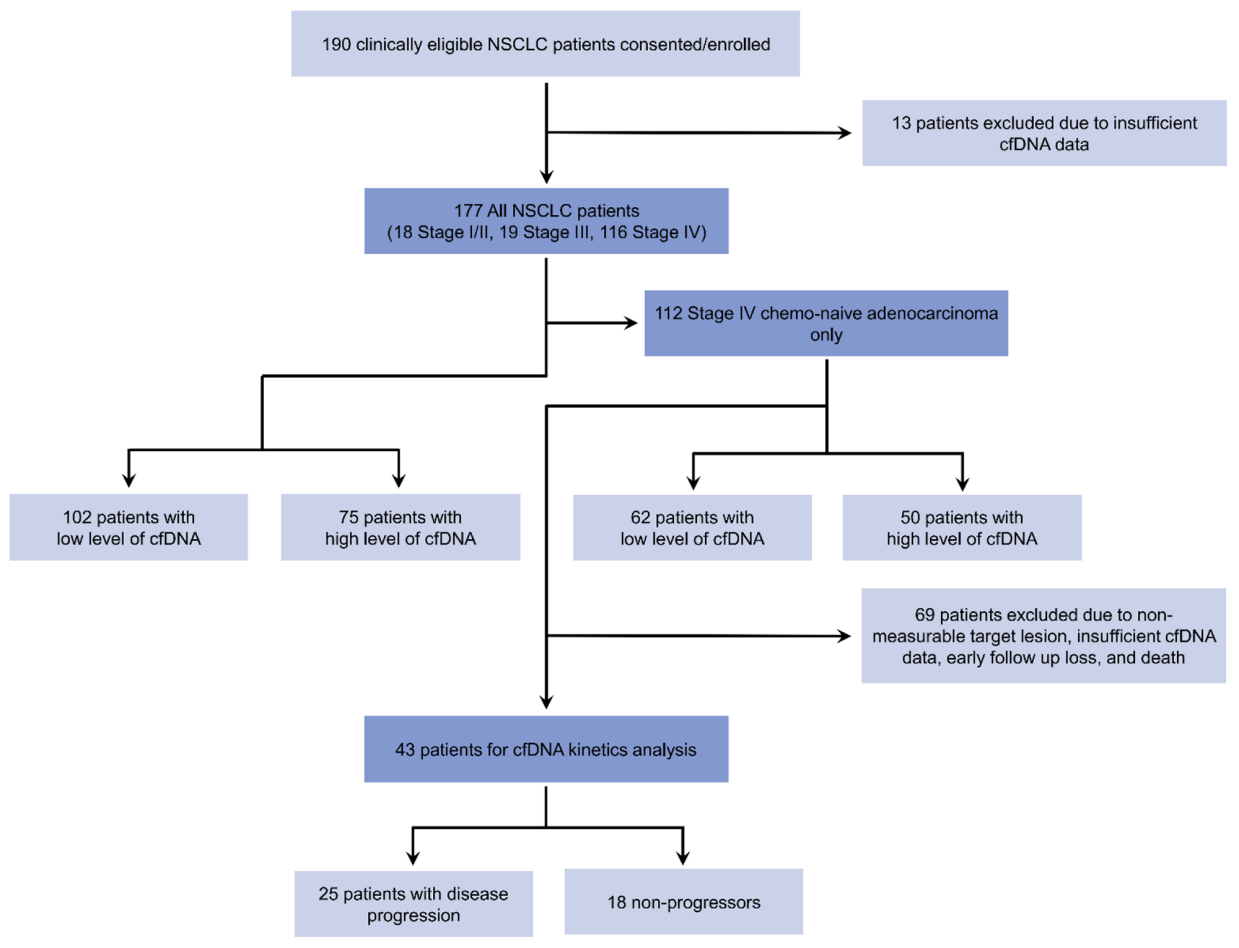
Study flow diagram cfDNA, cell-free DNA; NSCLC, non-small-cell lung cancer, ADC, adenocarcinoma.

We recruited 190 consecutive patients with NSCLC at the Korea University Anam Hospital and Guro Hospital from April 2006 to April 2017. All samples and medical records were irreversibly anonymized. Following the exclusion of patients with insufficient data, 177 patients with NSCLC were enrolled in a prospective biomarker trial to evaluate the prognostic value of cfDNA. Among them, 112 chemo-naive patients with stage IV adenocarcinoma were analyzed as the follow-up cohort. Of these 112 study subjects, 69 patients were excluded due to non-measurable target lesions, insufficient cfDNA data, loss to early follow-up, or death before response evaluation. Ultimately, 43 patients were available for the cfDNA kinetic analysis (25 patients [58%] with disease progression and 18 [42%] without disease progression). The details of the study selection process are presented in Figure 5.

The inclusion criteria were histopathologically confirmed NSCLC, age above 18 years, no previous cancer diagnosis within 5 years, and provision of written informed consent. The choice of systemic therapy, including platinum and/or non-platinum-based chemotherapy and/or tyrosine kinase inhibitors, was not limited for the analysis. The NSCLC histologic subtype was determined according to the World Health Organization classification [37]. The tumor node metastasis international staging system was used in accordance with the American Joint Committee on Cancer 8th edition guidelines [38]. The ECOG PS and Charlson Comorbidity Index were used to evaluate patients’ performance status and comorbidities [39, 40].

### Circulating cfDNA purification and quantification

Peripheral blood samples (10 mL) were collected from all patients prior to treatment and placed in serum separator tubes. Within 1 hour after collection, the samples were centrifuged for 10 min at 2000 × *g* at 20°C. The supernatants were transferred to microcentrifuge tubes, then centrifuged again for 5 min at 16,000 × *g* at 20 °C. Subsequently, the supernatants were stored at −80°C until use. cfDNA was isolated from serum aliquots (500 μL) by means of a QIAamp Circulating Nucleic Acid Kit (Qiagen, Hilden, Germany) with a QIAvac 24 Plus vacuum manifold, in accordance with the manufacturer’s instructions. After cfDNA extraction, the purity of the cfDNA was measured with an Agilent High Sensitivity DNA kit and a Bioanalyzer 2100 instrument (Agilent Technologies, Santa Clara, CA, USA). If needed, additional purification was performed with Agencourt AMPure XP (Beckman Coulter, Brea, CA, USA) to minimize DNA contamination. Subsequently, the circulating cfDNA concentration was quantified with a Qubit 2.0 Fluorometer and the Agilent High Sensitivity DNA kit (Agilent Technologies).

### Outcome assessment

All patients underwent follow-up for PFS and OS analysis. PFS was defined from the date of study inclusion to the first evidence of disease progression or death from any cause. OS was calculated from the date of study inclusion to death from any cause. For the response to systemic therapy, the SLDs of measurable target lesions were quantified via CT in accordance with the standard Response Evaluation Criteria in Solid Tumors version 1.1 [41]. CT scans were obtained at baseline, at 6 to 12 weeks after initiation, and then every 6 to 12 weeks. For the cfDNA kinetic analysis, consecutive paired blood collection was performed at baseline, within 2 weeks of baseline CT, and at response assessment within 2 weeks of the serial follow-up CT.

### Statistics

cfDNA data were expressed as the median and IQR. Correlations of baseline cfDNA concentrations with patient clinicopathologic characteristics were analyzed with the Mann-Whitney rank sum test and the Kruskal-Wallis test. ROC curves were constructed to evaluate the diagnostic capability (i.e., sensitivity, specificity and AUC) of the cfDNA concentration to discriminate stage IV from stages I/II/III. X-tile analysis [42] was used to define the best cfDNA concentration cut-off value (70 ng/mL) for survival prediction. For the X-tile analysis [42], two-thirds of patients were randomized as the training set, while the remaining one-third of patients comprised the validation set according to PFS and OS. Then, the optimal cut-off value was determined by training/validation methods. Details on the determination of the best cfDNA concentration cut-off value are described in Supplementary Figure 3.

Kaplan-Meier estimation was used to designate PFS and OS, and the log-rank test was used to determine significant differences. Cox proportional hazards analysis was used to further examine whether cfDNA levels were associated with survival after adjustment for covariates such as age, sex, ECOG PS, Charlson Comorbidity Index and clinical stage. The correlation between the percentage change in the cfDNA level and the percentage change in the SLD at the radiological first response was determined with Spearman’s rank test. Changes in the cfDNA concentration at different time points were estimated with the Wilcoxon signed rank sum test. Given the heterogeneity of tumor progression, CT radiological responses were categorized as disease progression and no progression, and further subdivided by the radiological response during treatment, in accordance with the Response Evaluation Criteria in Solid Tumors version 1.1 [41]. The intergroup percentage changes in the cfDNA concentration were assessed with the Mann-Whitney U-test or the Kruskal-Wallis test. A two-sided p value ≤ 0.05 was considered statistically significant. All statistical analyses were performed with SPSS version 24.0 (SPSS Inc., Chicago, IL, USA), X-tile software version 3.6.1 (Yale University, New Haven, CT, USA), R software version 3.2.2 (R Core Team, Vienna, Austria), or GraphPad Prism version 7.0 (GraphPad Software Inc., San Diego, CA, USA).

## SUPPLEMENTARY MATERIALS FIGURES



## Abbreviations

ADC: Adenocarcinoma
AUC: Area under the curve
CI: Confidence interval
cfDNA: Cell-free deoxyribonucleic acid
CT: Computed tomography
ECOG PS: Eastern Cooperative Oncology Group Performance Status
HR: Hazard ratio
IQR: Interquartile range
NSCLC: Non-small-cell lung cancer
OS: Overall survival
PFS: Progression-free survival
PR: Partial response
PD: Progression of disease
ROC: Receiver operating characteristic
SD: Stable disease
SLD: Sum of the longest diameter

## References

[1] Diamantis A, Magiorkinis E, Koutselini H (2009). Fine-needle aspiration (FNA) biopsy: historical aspects. Folia Histochem Cytobiol.

[2] Siegel R, Naishadham D, Jemal A (2013). Cancer statistics, 2013. CA Cancer J Clin.

[3] Shin A, Oh CM, Kim BW, Woo H, Won YJ, Lee JS (2017). Lung Cancer Epidemiology in Korea. Cancer Res Treat.

[4] Vanderlaan PA, Yamaguchi N, Folch E, Boucher DH, Kent MS, Gangadharan SP, Majid A, Goldstein MA, Huberman MS, Kocher ON, Costa DB (2014). Success and failure rates of tumor genotyping techniques in routine pathological samples with non-small-cell lung cancer. Lung Cancer.

[5] Huang WL, Chen YL, Yang SC, Ho CL, Wei F, Wong DT, Su WC, Lin CC (2017). Liquid biopsy genotyping in lung cancer: ready for clinical utility?. Oncotarget.

[6] Murtaza M, Dawson SJ, Tsui DW, Gale D, Forshew T, Piskorz AM, Parkinson C, Chin SF, Kingsbury Z, Wong AS, Marass F, Humphray S, Hadfield J (2013). Non-invasive analysis of acquired resistance to cancer therapy by sequencing of plasma DNA. Nature.

[7] Schwarzenbach H, Hoon DS, Pantel K (2011). Cell-free nucleic acids as biomarkers in cancer patients. Nat Rev Cancer.

[8] Jahr S, Hentze H, Englisch S, Hardt D, Fackelmayer FO, Hesch RD, Knippers R (2001). DNA fragments in the blood plasma of cancer patients: quantitations and evidence for their origin from apoptotic and necrotic cells. Cancer Res.

[9] Jiang T, Ren S, Zhou C (2015). Role of circulating-tumor DNA analysis in non-small cell lung cancer. Lung Cancer.

[10] Stroun M, Maurice P, Vasioukhin V, Lyautey J, Lederrey C, Lefort F, Rossier A, Chen XQ, Anker P (2000). The origin and mechanism of circulating DNA. Ann N Y Acad Sci.

[11] Jiang T, Zhai C, Su C, Ren S, Zhou C (2016). The diagnostic value of circulating cell free DNA quantification in non-small cell lung cancer: A systematic review with meta-analysis. Lung Cancer.

[12] Zhang R, Shao F, Wu X, Ying K (2010). Value of quantitative analysis of circulating cell free DNA as a screening tool for lung cancer: a meta-analysis. Lung Cancer.

[13] Ai B, Liu H, Huang Y, Peng P (2016). Circulating cell-free DNA as a prognostic and predictive biomarker in non-small cell lung cancer. Oncotarget.

[14] Cargnin S, Canonico PL, Genazzani AA, Terrazzino S (2017). Quantitative Analysis of Circulating Cell-Free DNA for Correlation with Lung Cancer Survival: A Systematic Review and Meta-Analysis. J Thorac Oncol.

[15] Gautschi O, Bigosch C, Huegli B, Jermann M, Marx A, Chasse E, Ratschiller D, Weder W, Joerger M, Betticher DC, Stahel RA, Ziegler A (2004). Circulating deoxyribonucleic Acid as prognostic marker in non-small-cell lung cancer patients undergoing chemotherapy. J Clin Oncol.

[16] Kumar S, Guleria R, Singh V, Bharti AC, Mohan A, Das BC (2010). Plasma DNA level in predicting therapeutic efficacy in advanced nonsmall cell lung cancer. Eur Respir J.

[17] Li BT, Drilon A, Johnson ML, Hsu M, Sima CS, McGinn C, Sugita H, Kris MG, Azzoli CG (2016). A prospective study of total plasma cell-free DNA as a predictive biomarker for response to systemic therapy in patients with advanced non-small-cell lung cancers. Ann Oncol.

[18] Pan S, Xia W, Ding Q, Shu Y, Xu T, Geng Y, Lu Y, Chen D, Xu J, Wang F, Zhao C, Huang P, Huang P (2012). Can plasma DNA monitoring be employed in personalized chemotherapy for patients with advanced lung cancer?. Biomed Pharmacother.

[19] Lee YJ, Yoon KA, Han JY, Kim HT, Yun T, Lee GK, Kim HY, Lee JS (2011). Circulating cell-free DNA in plasma of never smokers with advanced lung adenocarcinoma receiving gefitinib or standard chemotherapy as first-line therapy. Clin Cancer Res.

[20] Tissot C, Toffart AC, Villar S, Souquet PJ, Merle P, Moro-Sibilot D, Perol M, Zavadil J, Brambilla C, Olivier M, Couraud S (2015). Circulating free DNA concentration is an independent prognostic biomarker in lung cancer. Eur Respir J.

[21] Vinayanuwattikun C, Winayanuwattikun P, Chantranuwat P, Mutirangura A, Sriuranpong V (2013). The impact of non-tumor-derived circulating nucleic acids implicates the prognosis of non-small cell lung cancer. J Cancer Res Clin Oncol.

[22] Kwapisz D (2017). The first liquid biopsy test approved. Is it a new era of mutation testing for non-small cell lung cancer?. Ann Transl Med.

[23] cobas EGFR Mutation Test v2. 2016. available online: https://www.fda.gov/drugs/informationondrugs/approveddrugs/ucm504540.htm

[24] Lo YM, Zhang J, Leung TN, Lau TK, Chang AM, Hjelm NM (1999). Rapid clearance of fetal DNA from maternal plasma. Am J Hum Genet.

[25] van der Vaart M, Pretorius PJ (2008). Circulating DNA. Its origin and fluctuation. Ann N Y Acad Sci.

[26] Vargas AJ, Harris CC (2016). Biomarker development in the precision medicine era: lung cancer as a case study. Nat Rev Cancer.

[27] Fournie GJ, Courtin JP, Laval F, Chale JJ, Pourrat JP, Pujazon MC, Lauque D, Carles P (1995). Plasma DNA as a marker of cancerous cell death. Investigations in patients suffering from lung cancer and in nude mice bearing human tumours. Cancer Lett.

[28] Leon SA, Shapiro B, Sklaroff DM, Yaros MJ (1977). Free DNA in the serum of cancer patients and the effect of therapy. Cancer Res.

[29] Sirera R, Bremnes RM, Cabrera A, Jantus-Lewintre E, Sanmartin E, Blasco A, Del Pozo N, Rosell R, Guijarro R, Galbis J, Sanchez JJ, Camps C (2011). Circulating DNA is a useful prognostic factor in patients with advanced non-small cell lung cancer. J Thorac Oncol.

[30] Nygaard AD, Holdgaard PC, Spindler KL, Pallisgaard N, Jakobsen A (2014). The correlation between cell-free DNA and tumour burden was estimated by PET/CT in patients with advanced NSCLC. Br J Cancer.

[31] Sozzi G, Roz L, Conte D, Mariani L, Andriani F, Lo Vullo S, Verri C, Pastorino U (2009). Plasma DNA quantification in lung cancer computed tomography screening: five-year results of a prospective study. Am J Respir Crit Care Med.

[32] Dawson SJ, Tsui DW, Murtaza M, Biggs H, Rueda OM, Chin SF, Dunning MJ, Gale D, Forshew T, Mahler-Araujo B, Rajan S, Humphray S, Becq J (2013). Analysis of circulating tumor DNA to monitor metastatic breast cancer. N Eng J Med.

[33] Diehl F, Schmidt K, Choti MA, Romans K, Goodman S, Li M, Thornton K, Agrawal N, Sokoll L, Szabo SA, Kinzler KW, Vogelstein B, Diaz LA (2008). Circulating mutant DNA to assess tumor dynamics. Nat Med.

[34] Board RE, Williams VS, Knight L, Shaw J, Greystoke A, Ranson M, Dive C, Blackhall FH, Hughes A (2008). Isolation and extraction of circulating tumor DNA from patients with small cell lung cancer. Ann N Y Acad Sci.

[35] Kang Q, Henry NL, Paoletti C, Jiang H, Vats P, Chinnaiyan AM, Hayes DF, Merajver SD, Rae JM, Tewari M (2016). Comparative analysis of circulating tumor DNA stability In K3EDTA, Streck, and CellSave blood collection tubes. Clin Biochem.

[36] Elshimali YI, Khaddour H, Sarkissyan M, Wu Y, Vadgama JV (2013). The clinical utilization of circulating cell free DNA (CCFDNA) in blood of cancer patients. Int J Mol Sci.

[37] Travis WD, Brambilla E, Nicholson AG, Yatabe Y, Austin JH, Beasley MB, Chirieac LR, Dacic S, Duhig E, Flieder DB, Geisinger K, Hirsch FR, Ishikawa Y (2015). The 2015 World Health Organization Classification of Lung Tumors: Impact of Genetic, Clinical and Radiologic Advances Since the 2004 Classification. J Thorac Oncol.

[38] Amin MB, Edge SB, Greene FL, Byrd DR, Brookland RK, Washington MK, Gershenwald JE, Compton CC, Hess KR, Sullivan DC, Jessup JM, Brierley JD, Gaspar LE (2017). AJCC Cancer Staging Manual, 8th edition.

[39] Quan H, Li B, Couris CM, Fushimi K, Graham P, Hider P, Januel JM, Sundararajan V (2011). Updating and validating the Charlson comorbidity index and score for risk adjustment in hospital discharge abstracts using data from 6 countries. Am J Epidemiol.

[40] Sorensen JB, Klee M, Palshof T, Hansen HH (1993). Performance status assessment in cancer patients. An inter-observer variability study. Br J Cancer.

[41] Eisenhauer EA, Therasse P, Bogaerts J, Schwartz LH, Sargent D, Ford R, Dancey J, Arbuck S, Gwyther S, Mooney M, Rubinstein L, Shankar L, Dodd L (2009). New response evaluation criteria in solid tumours: revised RECIST guideline (version 1.1). Eur J Cancer.

[42] Camp RL, Dolled-Filhart M, Rimm DL (2004). X-tile: a new bio-informatics tool for biomarker assessment and outcome-based cut-point optimization. Clin Cancer Res.

