# Supplementary material for: Quantification of circulating cell-free DNA to predict patient survival in non-small-cell lung cancer
Source: Oncotarget. 2017 Oct 10;8(55):94417–30. doi: 10.18632/oncotarget.21769 (PMC5706884; doi:10.18632/oncotarget.21769)
Supplement: Supplementary file 1 [file oncotarget-08-94417-s001.pdf]

Quantification of circulating cell-free DNA to predict patient survival in non-small-cell lung cancer

SUPPLEMENTARY MATERIALS

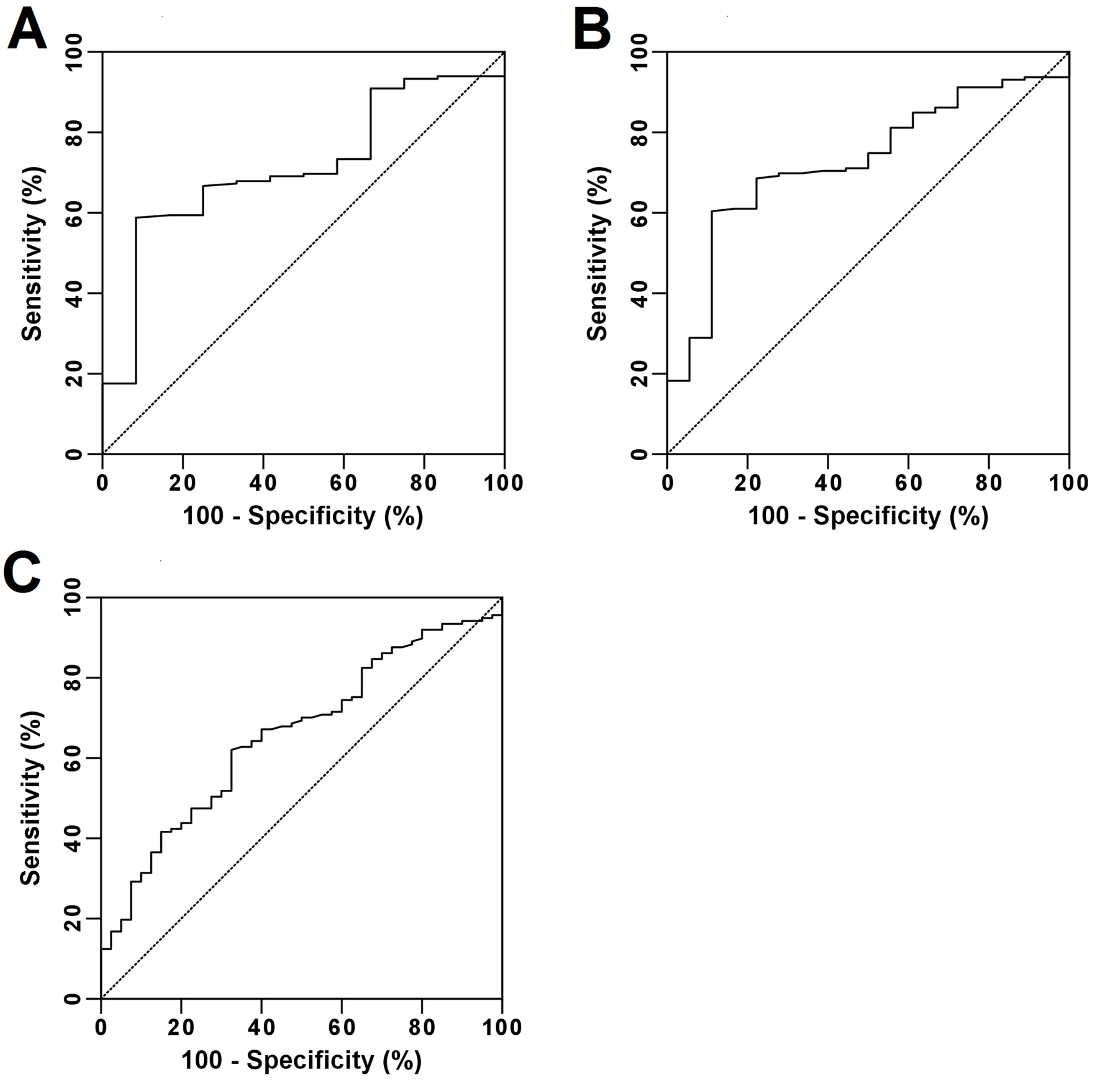

Supplementary Figure 1: ROC curve analysis of clinical stage prediction according to the circulating cfDNA concentration in patients with NSCLC (A) Stage I vs. stages II-IV; the AUC was 0.713 ($p < 0.014$; 95% CI: 0.587-0.839), and 50 ng/mL was determined as the cfDNA cut-off value for comparison. The sensitivity and specificity of the dichotomized cfDNA (≤ 50 vs. > 50 ng/mL) were 58.8% and 91.7%, respectively. **(B)** Stages I-II vs. stages III-IV; the AUC was 0.727 ($p < 0.002$; 95% CI: 0.621-0.834), and 50 ng/mL was determined as the cfDNA cut-off value for comparison. The sensitivity and specificity of the dichotomized cfDNA (≤ 50 vs. > 50 ng/mL) were 60.4% and 88.9%, respectively. **(C)** Stage I-III vs. stage IV; the AUC was 0.658 ($p < 0.002$; 95% CI: 0.568-0.748), and 50 ng/mL was determined as the cfDNA cut-off value for comparison. The sensitivity and specificity of the dichotomized cfDNA (≤ 50 vs. > 50 ng/mL) were 62.0% and 67.5%, respectively. AUC, area under the curve; ROC, receiver operating characteristic; cfDNA, cell-free DNA; NSCLC, non-small-cell lung cancer; CI, confidence interval.

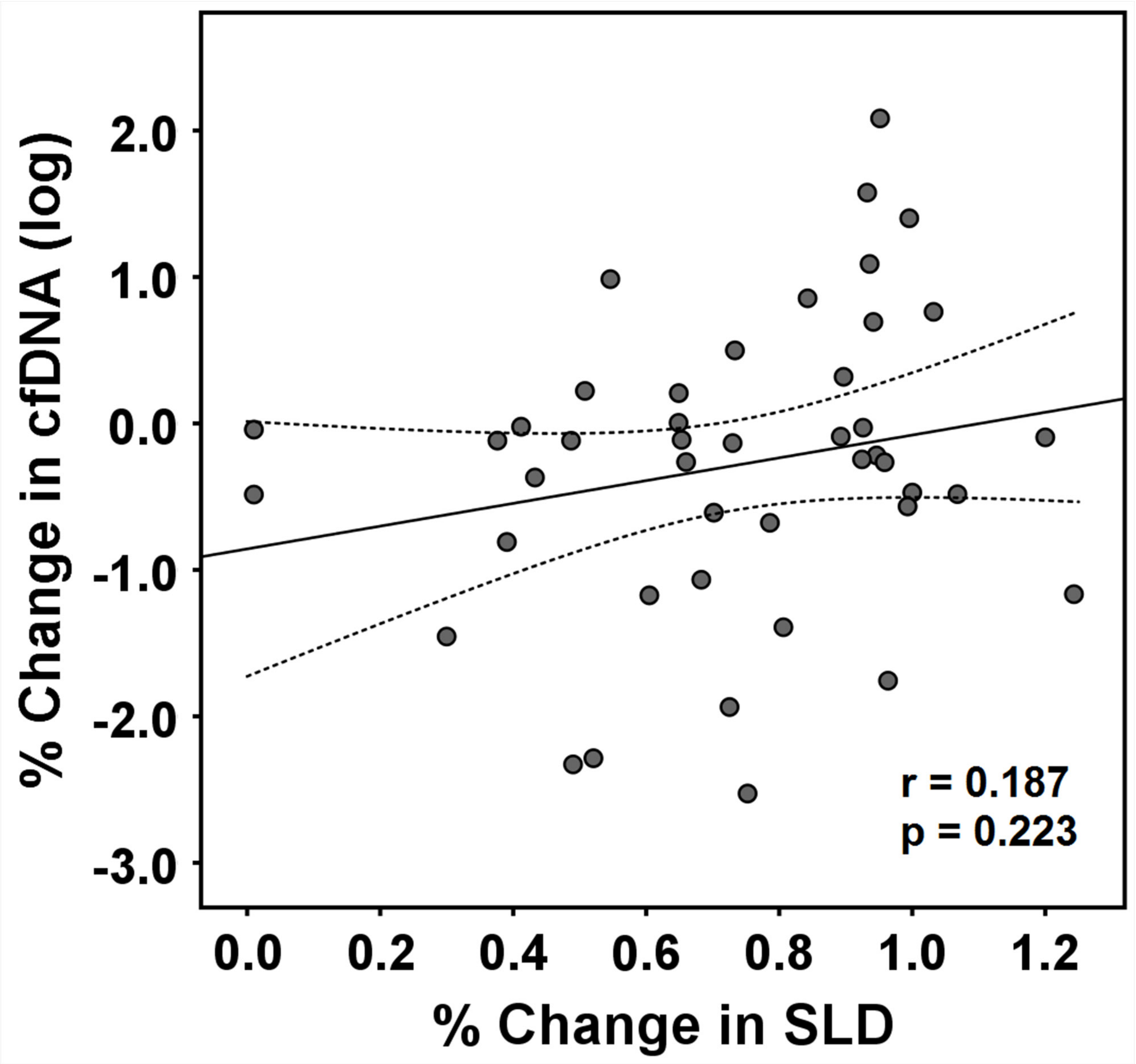

Supplementary Figure 2: Scatter plot for the correlation between the percentage change in the cfDNA concentration and the radiological response at assessment of first response. The solid line is the linear regression line, and the dashed lines are the 95% confidence intervals. cfDNA, cell-free DNA; SLD, sum of the longest diameters of measurable target lesions.

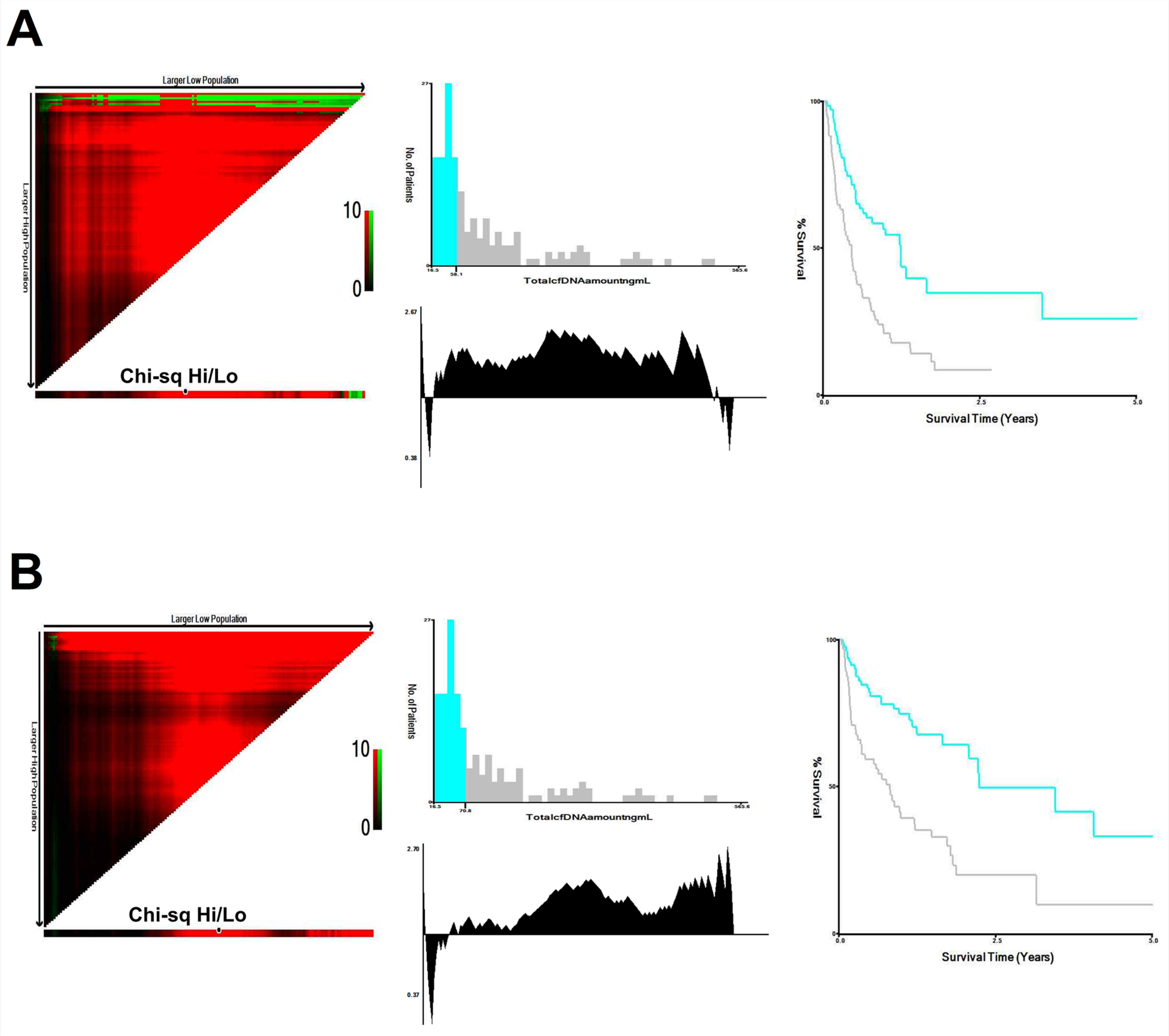

Supplementary Figure 3: X-tile analysis of PFS and OS data according to the cfDNA concentration in patients with NSCLC X-tile analysis of the prognostic significance of cfDNA was performed on patient data that were divided into matched training and validation sets (1:1 ratio). X-tile plots display the $\chi 2$ log-rank values with the cut-off value highlighted by the black/white circle in the rectangular X-tile plot (left panels), demonstrated in a histogram (middle panels) and a Kaplan-Meier plot (right panels). The optimal cut-off values were as follows: **(A)** PFS (cut-off value = 58.1 ng/mL, p min < 0.001), and **(B)** OS (cut-off value = 70.8 ng/mL, p min < 0.001). The small number of events favored the use of the OS cut-off value, which was close to the PFS cut-off value; thus, 70 ng/mL was determined as the optimal cut-off value for the cfDNA concentration. PFS, progression-free survival; OS, overall survival; cfDNA, circulating cell-free DNA; NSCLC, non-small-cell lung cancer.
